# Prevalence of Malnutrition in People with Dementia in Long-Term Care: A Systematic Review and Meta-Analysis

**DOI:** 10.3390/nu15132927

**Published:** 2023-06-28

**Authors:** Emma Perry, Karen Walton, Kelly Lambert

**Affiliations:** School of Medical, Indigenous and Health Sciences, University of Wollongong, Wollongong, NSW 2522, Australia; emmagperry99@gmail.com (E.P.); kwalton@uow.edu.au (K.W.)

**Keywords:** malnutrition, long-term care, geriatric, nutrition assessment, MNA, SGA, systematic review, meta-analysis

## Abstract

Dementia is a common syndrome in older people. Dementia alters eating behaviors, hunger and thirst cues, swallow function, ability to self-feed, and recognition and interest in food. There is significant variation in the reported prevalence of malnutrition among older people who live in long-term care. The aim was to conduct a systematic literature review and meta-analysis of the prevalence of malnutrition in those with dementia living in long-term care using a validated nutrition assessment tool. Scopus, Web of Science, CINAHL, and Medline were searched. A random effects model was used to determine the prevalence and risk of malnutrition. Data were retrieved from 24 studies. Most of the studies were from Europe or South Asia. The prevalence of malnutrition ranged from 6.8 to 75.6%, and the risk of malnutrition was 36.5–90.4%. The pooled prevalence of malnutrition in those with dementia in long-term care was 26.98% (95% CI 22.0–32.26, *p* < 0.0001, I^2^ = 94.12%). The pooled prevalence of the risk of malnutrition in those with dementia was 57.43% (95% CI 49.39–65.28, *p* < 0.0001, I^2^ = 97.38%). Malnutrition is widespread in those with dementia living in long-term care. Further research exploring malnutrition in other industrialized countries using validated assessment tools is required.

## 1. Introduction

Dementia is a syndrome associated with chronic progressive degeneration of the brain [1]. Dementia can impact memory, thinking, orientation, comprehension, and judgement, due to impairment in the higher cortical functions of the brain [1]. Many causes can be attributed to dementia, most notably Alzheimer’s disease (AD) contributing to 60–80% of cases, cerebrovascular disease leading to vascular dementia contributing to 5–10% of cases, and dementia with Lewy bodies contributing to 5% of cases [2]. The prevalence of dementia in those aged 60 years or more is between 5 and 7% [3,4]. Globally, this is projected to equate to ~66 million people with dementia in 2030, and the prevalence is anticipated to double every 20 years, reaching 78 million by 2030 and 139 million by 2050 [4,5]. The incidence of dementia doubles with every 6.3-year increase in age from 3.9 per 1000 person-years at age 60–64 years, up to 104.8 per 1000 person-years at age 90+, which is further amplified by the increase in population ageing worldwide [4].

Irrespective of the type of dementia or cause, food intake can be significantly altered due to the complex nature involving many physiological, pathological and psychological factors [4,6]. In the early stages of dementia, altered food intakes or changes may not be apparent or troublesome to the person with dementia or their care-givers [4,6]. However, in advanced and severe stages, people with dementia may lose the ability to recognize their hunger and thirst cues, as well as develop the inability to self-feed due to apraxia or the visuospatial dysfunction that accompanies advanced dementia [7]. Other factors contributing to poor oral intake include food disinterest or avoidance, difficulty recognizing familiar objects and confusion, reliance on others for eating assistance, and dysphagia, where in severe AD, brain damage can significantly decrease one’s ability to swallow [1,2].

As dementia advances, so does the requirement for assisted living due to their behavioral and psychological changes, including wandering, progressing cognitive impairment, and difficulties partaking independently in activities of daily living (ADL) [8]. According to the Australian Federal Government, 71% of those with dementia require high levels of care and require eating assistance. This highlights the major impact dementia has on an individual’s oral intake and risk of malnutrition [8]. Between 2015 and 2020, half (54%) of the 244,000 Australians living in permanent long-term care facilities had a diagnosis of dementia [8].

Malnutrition is ‘a subacute or chronic state of nutrition, in which a combination of varying degrees of under- or overnutrition and inflammatory activity has led to changes in body composition and diminished function’ [9]. Studies of malnutrition in long-term care facilities have found the prevalence or risk of malnutrition to be high. A cross-sectional study conducted in Spain found 61.9% of elderly patients were undernourished according to the MNA, and a Taiwanese cross-sectional study using the MNA-SF found that 90.4% residents were categorized ‘at risk’ [10,11].

Unfortunately, malnutrition is often undetected and neglected in long-term care facilities due to a lack of malnutrition screening and inadequate dietetic staffing. Biomarkers such as serum albumin and transferrin are unreliable assessment methods as they are influenced by other confounding factors such as inflammation, infection and the presence of co-morbidities [12]. Body mass index (BMI) is also often used for the assessment of malnutrition. However, this is a cross-sectional measure and consequently does not identify weight loss over time. Moreover, a high BMI does not indicate a lack of malnutrition [12]. A cross-sectional study in Australia determined that 34% of participants identified as ‘at risk’ according to the MNA-SF had a BMI that was >25 kg/m^2^, which underscores the necessity for using a validated assessment tool to diagnose malnutrition [13]. Several validated nutrition assessment tools can be used to determine nutritional status in older adults, which include the Mini Nutritional Assessment (MNA), Mini Nutritional Assessment—Short Form (MNA-SF), Subjective Global Assessment (SGA), and Patient Generated—Subjective Global Assessment (PG-SGA) [14]. Studies using these tools in the long-term care population often exclude those with dementia, despite the World Health Organisation declaring dementia as a public health priority in the Mental Health Gap Action Program in 2011 [1,15,16,17,18]. At present, no systematic literature review has been conducted to synthesize findings on this crucial topic. Therefore, the aim of this systematic literature review was to synthesize the existing evidence and determine the prevalence of malnutrition in those living with dementia in residential long-term care using validated nutrition assessment and screening tools.

## 2. Materials and Methods

The question of interest was “in people diagnosed with dementia (population) who are living in residential long-term care (context), what is the prevalence of malnutrition (diagnosed using a validated nutrition assessment tool such as the MNA [19], PG-SGA [20], SGA [21], or MNA-Short Form (MNA-SF) [22]) (concept)”. The study protocol was registered with PROSPERO (CRD42022314860). This systematic literature review was reported according to the Preferred Reporting Items for Systematic Reviews and Meta-Analyses (PRISMA) 2020 checklist [23].

### 2.1. Information Sources

The search used the terms (Malnutrition OR malnourished OR undernutrition OR undernourished OR “protein-energy malnutrition” OR sarcopenia OR “risk of malnutrition” OR “weight loss” OR “prevalence of malnutrition” OR “nutritional status”) AND (“subjective global assessment” OR SGA OR “patient generated subjective global assessment” OR “PG-SGA” OR “mini nutritional assessment” OR MNA OR “mini nutritional assessment short form” OR MNA-SF OR “nutrition assessment” OR “geriatric assessment” OR “malnutrition screening”) AND (“residential aged care” OR “aged care” OR “long term care” OR “nursing home” OR “home for the ag*” OR “housing for the elderly” OR “skilled nursing facility” OR “assisted living” OR “residential care” OR “geriatric institution*”) as Medical Subject Heading terms and keywords as per the database. The search terms had been identified by analyzing keywords in similar studies. The electronic databases Scopus, Web of Science, CINAHL, and Medline were searched on 24 April 2022 with no restrictions. The search terms were developed by one author (E.P.) in conjunction with a specialist librarian and revised by two authors of the research team (K.W. and K.L.). The reference lists of relevant studies on similar topics of dementia, malnutrition and long-term care facilities were also searched and a search for unpublished reviews on PROSPERO was conducted. The search strategies for each database are listed in Supplementary Table S1.

### 2.2. Study Selection

The search was conducted independently by one author (E.P.) and uploaded to Covidence software [24] where duplicates were removed. Title and abstract screening were completed by two authors (E.P. and K.L.) using the eligibility criteria and conflicts were sent to full-text review. Full-text review was completed independently by three authors (E.P., K.L. and K.W.) and any uncertainties were discussed between the three authors (E.P., K.L. and K.W.).

To be eligible for inclusion, studies were required to be primary research studies where malnutrition was assessed using a validated nutrition assessment tool, were conducted in long-term care or equivalent, and residents must have been diagnosed with dementia or cognitive impairment using relevant assessment tools or with a confirmed diagnosis reported in their medical record. Studies were excluded if they assessed malnutrition using non validated measures (such as albumin or BMI), reported for those without a probable diagnosis of dementia using a validated tool, including those with acute delirium, and studies not in English. Studies were also excluded if the outcome of interest, specifically the number of individuals with malnutrition and dementia, was unable to be determined or was not reported.

### 2.3. Data Extraction and Summary Measures

Data extraction was completed independently by one author (E.P.) in Microsoft Excel (version 16.65, 2022) [25] and a second author (K.L. or K.W.) reviewed each extraction for data completeness. Information extracted from the studies included participant information (mean age, mean time since admission), study demographics (author, year, country, study type), dementia information (the tool used for diagnosis and mean time since diagnosis), and malnutrition data (assessment tool used, who the assessment was conducted by, and prevalence or risk of malnutrition). All information gathered on the prevalence or risk of malnutrition was collected as a percentage of the population with dementia, and when not presented in this format, one of the authors (E.P.) calculated the percentage as appropriate for consistency in the presentation of results. Where data were not available in a study for an outcome of interest, one of the authors (E.P.) wrote ‘Not stated’ and no attempts were made to contact the publishers of the study.

### 2.4. Quality Appraisal

The Academy of Nutrition and Dietetics Criteria Checklist for Primary Research was used to assess the quality of each study [26]. The tool uses 10 questions to assess the quality of each study including the research question, selection of subjects free from bias and comparable, withdrawals, blinding, interventions and intervening factors, outcomes, statistical analysis, conclusions and funding and sponsorships by classifying answers into Yes, No, Unclear or Not applicable (N/A). Studies are then classified as negative, neutral or positive depending on final outcomes. The risk of bias was completed in duplicate (E.P. and K.W. or K.L.) independently and discrepancies were resolved by consensus between the authors.

### 2.5. Results Synthesis

Data on the total number of participants with dementia, and the prevalence of malnutrition and/or those at risk of malnutrition were calculated and inserted into Microsoft Excel (Version 16.65, 2022). Data were then exported into MedCalc (version 20.11) [27] to conduct the meta-analysis. Statistical significance was set at *p* < 0.05. The I^2^ statistic was used to evaluate and indicate degree of variance between studies and study heterogeneity, with a higher score closer to 100% indicating higher heterogeneity between the studies. A random effects model was used due to the diversity of studies globally and was determined to be better able to capture the true prevalence of malnutrition. Publication bias was assessed using a funnel plot and Egger’s test and the DerSimonian and Laird method was used for between study heterogeneity for rates of malnutrition.

## 3. Results

**Figure 1:**
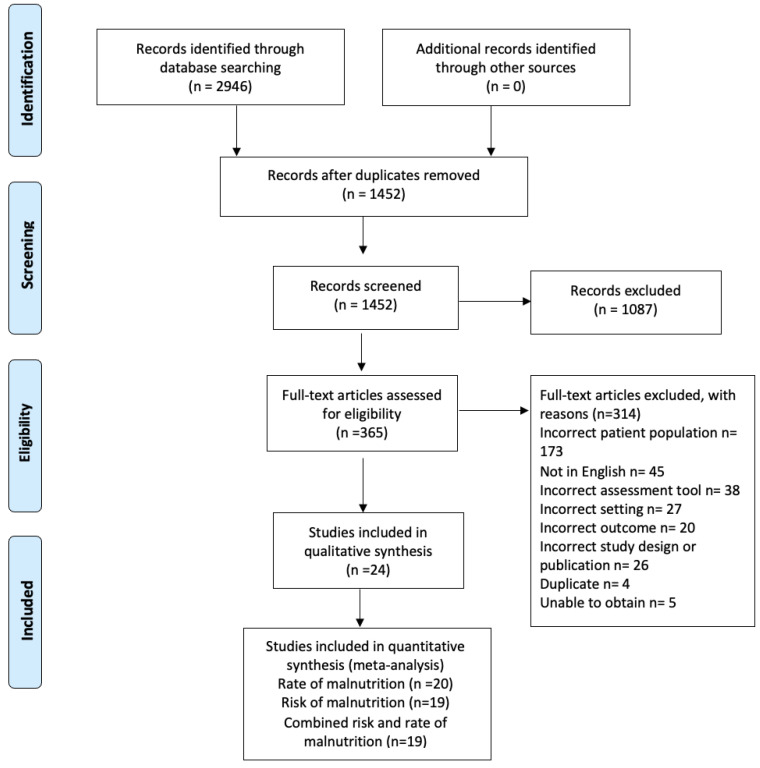
PRISMA 2020 flow diagram on included studies [23].

The initial search yielded 2946 studies. After the removal of 1494 duplicates, 1452 studies were screened via their title and abstract. This resulted in 365 full-text articles assessed for eligibility, with 24 studies included for analysis. Studies that did not specifically report on the number of those with dementia [28,29] were excluded. See Figure 1 for the PRISMA flow chart. Fifteen studies were cross sectional [11,30,31,32,33,34,35,36,37,38,39,40,41,42,43], three were randomized control trials [44,45,46], two were prospective cohort studies [47,48], two were observational studies [49,50], and two were pre–post intervention studies [51,52].

### 3.1. Participants

**Figure 2:**
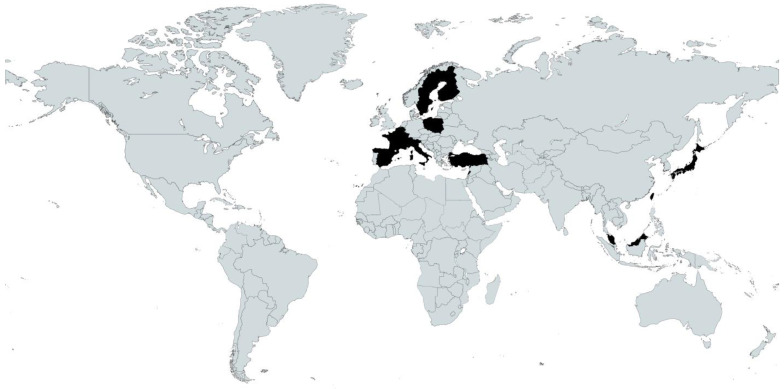
Locations of included studies in black. Map created with Mapchart.com.

A total of 8775 participants with dementia were included in the review ranging from 2 to 2379 participants per study [37,40] (Table 1). The geographic distribution of studies was spread across two continents, with 6 studies conducted in Asia (Taiwan [10,48], Turkey [31,36], Japan [50] and Malaysia [42]); and 18 studies conducted in Europe (Finland [37,38,40,51], France [32,45,46], Italy [30,49], Poland [39,52], Spain [11,44], Belgium [41], Lebanon [35], Sweden [47], Germany [43] and Switzerland [34]). See Figure 2 for a map of the geographic distribution of the included studies [53].

MNA [19] and MNS SF [54] have been validated for use in adults ≥ 65 years. SGA has been validated for use in adults [21]. MUST has been validated for use in inpatient and outpatient adults and has good agreement with the MNA and SGA [55].

Ten studies reported the mean age of those with dementia, with the lowest mean age being 74.5 ± 7.68 years [39] and highest mean age being 86.5 ± 6.1 years [44], whilst fourteen studies did not state the mean age. Nine studies reported on gender distribution with the lowest percentage of females at 59% [10] and the highest at 100% [40,52], but no studies reported on the prevalence of malnutrition between genders. Two studies reported on the mean time since dementia diagnosis ranging from 49.1 ± 24 months [44] to 51.1 ± 26.9 months [10]. Two studies reported on the mean time since admission to a long-term care facility ranging from 1.7 ± 1.6 years [44] to 2.4 ± 1.3 years [36].

Six studies used medical records to obtain the dementia diagnosis of the patients [10,37,38,43,47,50]; however, the tool used was not stated. Five studies did not state how the dementia diagnosis was made [32,36,41,45,51], and one study stated that the dementia was pre-existing [31].

### 3.2. Assessment Tools Used

A wide range of tools were used for the diagnosis of dementia (Table 1). These included the Mini Mental State Examination (MMSE) [11,35,51], Abbreviated Mental Test Score (AMTS) [52], Global Deterioration Scale (GDS) [39], and mini-cog test [42]. A number of publications also used multiple tools for the diagnosis of dementia, including the Diagnostic and Statistical Manual of Mental Disorders (DSM) and National Institute of Neurological and Communicative Disorders Alzheimer’s Disease and Related Disorders Association [48], the GDS and Pfeiffer test [30], the DSM and MMSE [46], medical records or interviews [49], the National Institute of Neurological and Communicative Disorders and Stroke (NINCDS) and the Alzheimer’s Disease and Related Disorders Association (ADRDA) criteria [44]. One study used three methods including the MMSE, Clinical Dementia Rating (CDR) scale and Consortium to Establish a Registry for Alzheimer’s Disease [34].

Several validated nutrition assessment or screening tools were identified as in use. This included the Mini Nutritional Assessment (MNA) in fifteen studies [11,34,35,36,37,38,39,42,46,49,51,52], and the Mini Nutritional Assessment- Short Form (MNA-SF) [41,48,50]. Several studies used multiple tools including the MNA and MNA-SF [10,31,32,47], the MNA-SF and Patient Generated Subjective Global Assessment (PG-SGA) [40] and one study used the Malnutrition Universal Screening Tool (MUST) [30].

Nine studies did not state who conducted the nutrition assessment [11,34,36,39,44,46,48,50]. In those studies who documented this, eight studies reported that nurses conducted the assessment [30,31,37,38,40,47,51,52], two studies had healthcare providers (nurse, general practitioner or other) [41,42], two had dietitians conduct the assessment [32,49], two had researchers conduct the assessment [10,45] and one had a dentist conduct the assessment [43].

### 3.3. Prevalence of Malnutrition

**Figure 3:**
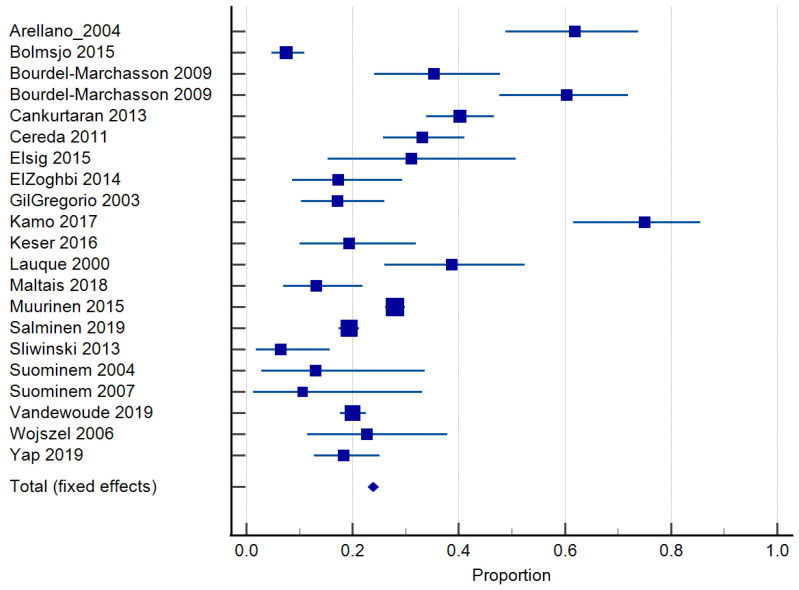
Forest plot of prevalence of malnutrition using random effects model. Q = 340.02, DF 20, *p* < 0.0001, I^2^ = 94.12%, 95% CI for I^2^ = 92.22–95.55. Publication bias—Egger’s Test, intercept = 0.99, 95% CI = −1.91–3.89, *p* = 0.48. Data from references [11,31,32,34,35,36,37,38,39,40,41,42,44,45,46,47,49,50,51,52].

**Figure 4:**
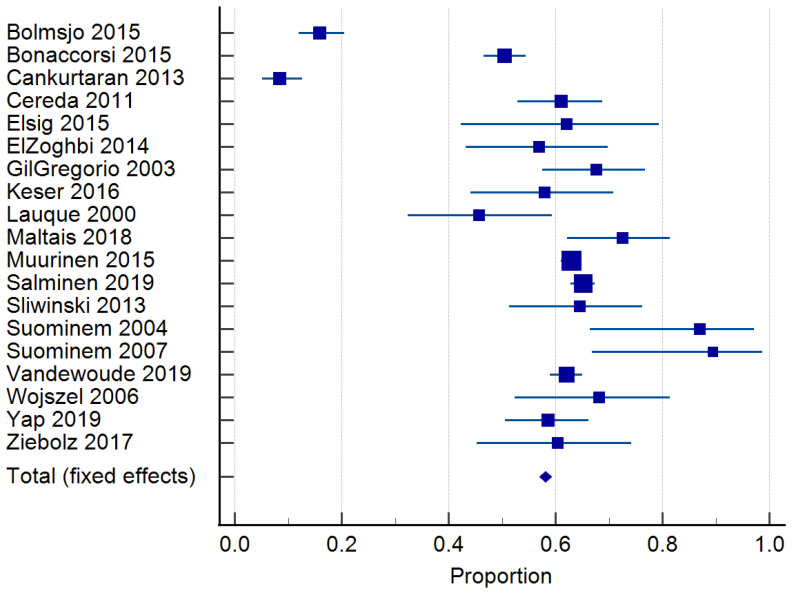
Forest plot of risk of malnutrition using random effects model. Q = 686.98, DF 18, *p* < 0.0001, I^2^ = 97.38%, 95% CI for I^2^ = 96.71–97.92. Publication bias—Egger’s test, intercept = −1.49, 95% CI = −6.52–3.26, *p* = 0.52. Data from references [30,31,34,35,36,37,38,39,40,41,42,43,44,45,46,47,49,51,52].

**Figure 5:**
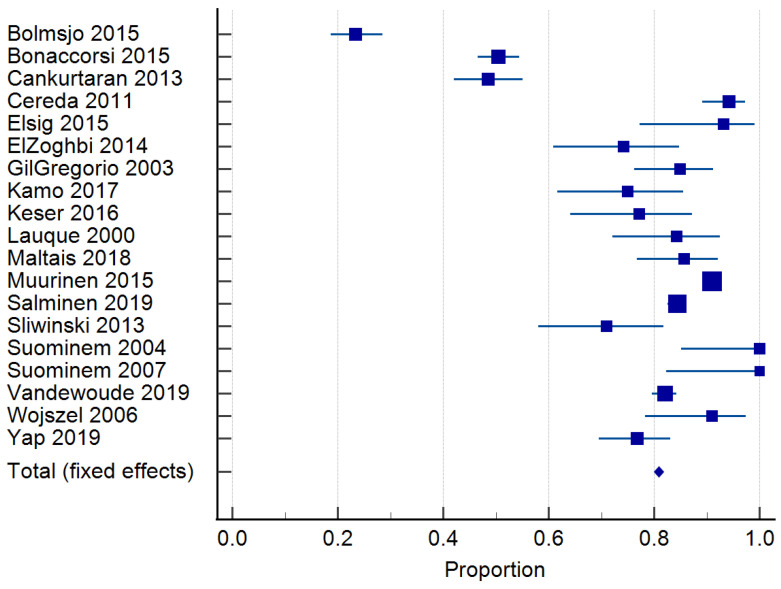
Forest plot of combined prevalence of those malnourished and at risk of malnutrition using random effects model. Q = 1142.78, DF = 18, *p* < 0.0001, I^2^ = 98.42%, 95% CI = 98.09–98.70. Publication bias—Egger’s test, intercept = −2.43, 95% CI =−8.53–3.67, *p* = 0.41. Data from references [30,31,34,35,36,37,38,39,40,41,42,44,45,46,47,49,50,51,52].

Twenty-four studies reported on the prevalence of malnutrition (Table 1). Prevalence ranged from 6.8 to 75% [46,50]. The risk of malnutrition ranged from 36.5 to 90.4% [10,11]. One study classified malnutrition risk according to dementia severity and found that 35.3% of those with mild dementia were malnourished according to the MNA and MNA-SF, compared to 60.3% with severe dementia [32]. In total, 20 studies [10,30,31,33,34,35,36,37,38,39,40,41,43,44,45,46,48,49,50,51] of 6769 residents were included in the meta-analysis of malnutrition and 19 studies [29,30,33,34,35,36,37,38,39,40,41,42,43,44,45,46,48,50,51] of 7202 residents for the risk of malnutrition. The pooled prevalence of malnutrition in those with dementia was 26.98% (95% CI 22.0–32.26, *p* < 0.0001, I^2^ = 94.12%) (Figure 3). The pooled prevalence for the risk of malnutrition in those with dementia was 57.43% (95% CI 49.39–65.28, *p* < 0.0001, I^2^ = 97.38%) (Figure 4). The pooled prevalence of those considered both at risk and malnourished with dementia was 79.66% (95% CI 70.86–87.22, *p* < 0.0001, I^2^ = 98.42%) (Figure 5) [29,30,33,34,35,36,37,38,39,40,41,43,44,45,46,48,49,50,51]. The results of Egger’s test indicated there was no publication bias for the studies included regarding the prevalence of malnutrition (*p* = 0.48), risk of malnutrition (*p* = 0.52), and combined prevalence and risk of malnutrition (*p* = 0.41).

### 3.4. Quality Assessment

Most of the 24 studies were rated as positive (18 studies) [10,30,31,34,35,36,37,38,40,41,42,43,45,46,47,49,50,52], or neutral (6 studies) [11,32,39,44,48,51]. Of the studies classified as neutral, five had potential bias in participant selection [11,32,39,44,48], and four had unclear bias due to funding or sponsorship [11,32,44,48]. Due to the nature of data collection through nutrition assessments, blinding was only reported in two studies [43,46], with two studies reporting that blinding was not used [42,45,52], three were unclear if blinding was used [41,42,51] and eighteen reported not applicable [10,11,30,31,32,34,35,36,37,38,39,40,44,47,48,49,50]. The most common study designs used were non controlled trials [10,11,30,31,32,34,35,36,37,38,39,40,41,42,43,51,52]; cohort studies [47,48,49,50] and two randomized trials [44,45,46]. Supplementary Table S2 contains further details of the quality assessment.

## 4. Discussion

To our knowledge, this is the first systematic literature review reporting the prevalence of malnutrition in those with dementia living in long-term care using established, validated nutrition screening or assessment tools. Analysis of the 24 eligible studies suggest that the prevalence and risk of malnutrition in this population and setting is high, with a pooled prevalence of malnutrition of 26.98%, risk of malnutrition at 57.43%, and the combined pooled prevalence was 79.66%.

These high rates of malnutrition in those with dementia are concerning due to the life expectancy increasing worldwide, thus an increased likelihood of individuals developing dementia as they age [3]. Ten studies in our review stated the age of the patients which ranged from the lowest mean age of 74.5 ± 7.68 years [39] to the highest mean age of 86.5 ± 6.1 years [44]. An analysis from the Health and Retirement Study (HRS) and determined the mean age for incident dementia was 83.72 years ± 5.49 [56]. Moreover, as one ages, so does the likelihood of dementia progression, with advanced stages of dementia diagnosed in only 6.2% of 65–68-year-old adults but increasing to 24.2% by age 95 [3,57]. In our review, the mean ages are lower than that of incident dementia, thus it is likely the determined prevalence of malnutrition is underestimated. Studies often exclude those with severe dementia due to their limited cognitive ability, communication and ethical issues surrounding consent [58,59,60]. At this stage of life, it can be unnecessary to conduct a nutrition assessment, as any intervention provided should be focused on improving quality of life and intake of preferred foods rather than a nutrition intervention [58,59,60].

Our results are similar to a systematic review using only the MNA [61]. Cereda et al. determined the pooled prevalence of malnutrition in those with dementia living in long-term care was 15.2% (95% CI: 10.9–19.4) and the risk of malnutrition was 49.2% (95% CI: 43.9–54.5 CI) with the study determining higher rates of malnutrition are present in those with higher levels of dependence and care requirements [61]. Similarly, an analysis of cognitive impairment in residents in long-term care in Italy found 0% of residents had a normal nutrition status, 33.3% were at risk and 66.7% were malnourished according to the MNA [62].

Of interest in this review was the gender distribution in the included studies. Nine studies reported on gender distribution, with the lowest percentage of females at 59% [10] and highest at 100% [40,51], but interestingly, no studies specifically reported on the prevalence or risk of malnutrition between the genders. One study has suggested that women are 45% more likely to be malnourished than men and have a significantly lower MNA [63,64]. The increased prevalence of dementia in females may be the result of the higher life expectancy of women, particularly as women with dementia have a 0.5 year longer life expectancy than men [65]. Moreover, there is a disproportionate distribution of women compared to men accessing care services, particularly in long-term care with an Australian study reporting 65% of people in long-term care are women [66].

Many factors contribute to the high prevalence of malnutrition in those with dementia. This includes knowledge that those with AD often have an increased resting energy expenditure (REE) and feeding difficulties [67,68]. An analysis of energy intake and resting energy expenditure (REE) in those with AD and cognitive impairment determined that AD patients had a significantly higher REE than those without AD (1704 ± 41 and 1754 ± 47 vs. 1569 ± 34 kcal/day, *p* < 0.05) [67]. It is important to note that feeding difficulties are reported to affect 44.6% of those with dementia [68,69]. Furthermore, co-morbidities particularly dysphagia are present in 7–40% of those in long-term care [70]. One study in our review analyzed the presence of dysphagia in those living in long-term care in Italy. Although not specific for those with dementia, they found 58.1% of residents had dysphagia and 36.8% were considered at medium risk for malnutrition using the MUST [30]. Similarly, a high rate of malnutrition (45%) was also found in those with chewing problems and dementia [71].

Screening and diagnosis of malnutrition can be conducted using a range of validated tools. The MUST and MNA-SF can be used for screening and the MNA and PG-SGA for assessment. The MNA-SF and MNA are validated for older adults only [14,72]. Nineteen studies in our review used the MNA, which the ESPEN Guidelines state it is recommended for use in the elderly and in long-term care [72]. In a separate analysis of nutrition tools used in long-term care in Australia, the tools used most frequently were the MNA at 32%, followed by the MUST at 15% [73]. The MNA is useful in detecting the likelihood of undernutrition in frail elderly populations and can detect malnutrition in the early stages before severe consequences take place, including severe weight loss [20,72]. Overall, the MNA has good reliability; however, it includes questions related to intake, which may elicit unreliable responses in a person with cognitive impairment [14,72]. Our review found that the MNA-SF was used in eight studies, which can be used in older adults with high sensitivity in detecting malnutrition but poor overall validity [74,75]. However, only two studies in our review used the MNA-SF exclusively and we did not find that the results differed from studies using other tools. One study used the PG-SGA, which is a modified SGA nutrition assessment that allows for an assessment of more factors, such as those impacting eating and functional impairment [14]. Although the PG-SGA is widely accepted as a tool for use in oncology patients, it is still valid and sensitive in detecting malnutrition, and can be useful for screening, assessment, triaging and monitoring [20]. Lastly, one study used the MUST, which, according to the ESPEN Guidelines, is a tool recommended for nutrition screening in the community that has a high level of reliability; however, it can be used in all health care settings [30,72]. An analysis of the MNA and MUST nutrition tools in long-term care found the MNA could only be applied to 94% of residents, whereas the MUST could be applied to 99% due to the alternative measures it allows for, catering for those with disabilities [76]. However, the MUST has not been adapted to older people as it does not account for their recommended higher BMI range, whilst the MNA is specifically designed for the elderly [76].

Interestingly, no study in our review compared malnutrition rates in those with dementia in long-term care to those in the community; thus, we cannot compare nutritional status between locations. One review found the prevalence of malnutrition in long-term care to be the highest at 21.6% compared to only 9.2% in the community [77]. This is in line with the results determining that only 3.1% of community dwelling older adults were malnourished in contrast to 17.5% in long-term care [61]. This high discrepancy between the locations amplifies the importance of monitoring and intervening in those malnourished living in long-term care facilities.

An unexpected finding of this study was the limited number of studies and limited distribution of studies globally. Three quarters of the studies were conducted in Europe and 25% of the studies were conducted in South Asia. This review is therefore not able to provide insight into the issue of malnutrition in the United Kingdom, North America, South America, Africa, or Australasia. The World Alzheimer’s Report in 2015 found that the highest prevalence of AD globally was in East Asia and Western Europe and determined that the distribution of new dementia cases was 49% in Asia (including Australia), 25% in Europe, 18% in the Americas and 8% in Africa [4]. The average prevalence of dementia in countries that are members of the Organisation for Economic Co-operation and Development (OECD) is 15.3 per 1000 population [8]. The highest prevalence of dementia was apparent in Japan at 24.8 per 1000 [8]. One study from our review was conducted in Japan and found high results of malnutrition at 75.6%, whilst only 24.4% were at risk or well-nourished [50]. These high results can also be observed in a 2022 cross-sectional study on malnutrition conducted in long-term care in Japan using the MUST and Global Leadership Initiative on Malnutrition, whereby 34.3% of those with dementia had severe malnutrition [78]. This is explained by Japan having the most rapidly ageing population, with the proportion of those aged ≥65 years projected to reach 40% by 2040–2050 [78]. Our review found that the lowest prevalence of malnutrition was in France, as only 6.8% of long-term care residents were considered to be malnourished [46]. France has instigated policies to deliver and prioritize the planning of health care services for older people through health professionals including dietitians being employed to work in care homes, which may explain the lower prevalence of malnutrition in this study due to the provision of care that a dietitian can provide [79]. As dementia is most associated with the greatest growing and ageing population group, more studies are required globally to determine the prevalence of malnutrition in long-term care facilities and intervene with public health initiatives.

It is important to note that dietitians are essential staff in a long-term care facility, but unfortunately, only two studies used dietitians to conduct the nutrition assessment, and nine studies did not state who conducted the assessment. Nurses play a crucial role in long-term care facilities; however, they are likely to need more training in the prevention, detection and management of malnutrition in residents with dementia. One study examining nurses’ ability to detect malnutrition found an alarming discrepancy between malnutrition prevalence according to nurses (11% of residents) compared to 32.9% using objective criteria for malnutrition [80]. Similarly, using the MNA, nurses only identified 15.2% of patients to be malnourished, whilst the MNA scores suggested that 56.7% were malnourished [81]. This may be strongly impacted by perceptions of BMI, as only 2% of nurses correctly identified those who were malnourished with an MNA score < 17 but a BMI >20 [81]. One Australian study of nurses’ nutrition knowledge in long-term care found self-ranked mean nutrition knowledge scores of 4.67/10 for nurses and only 38% of staff could identify reasons for increased protein and energy requirements [82]. These studies suggest that regular involvement of dietitians to identify and commence early medical nutrition therapy is critical. An Australian study of dietetic employment in long-term care facilities found that 78% of long-term care facilities employed dietitians, but this was on a predominately ad hoc (39%), casual (30%), and part-time (<10 h a week, 16%) basis [73].

There are several limitations to this systematic literature review. Studies were restricted to articles in English, which meant that eligible studies in other languages may have been excluded. The restriction to scientific databases meant some relevant studies may not have been found, and we did not contact the authors for further information; therefore, potential data may have been excluded. The high level of heterogeneity between studies may be the result of differing tools used to assess the outcome of malnutrition. While a random effects model was used, this heterogeneity indicates there is some uncertainty in the results. Dementia diagnoses are known to be under recorded in low- and middle-income countries [3], which provides uncertain overage of the evidence base regarding malnutrition in dementia. Differing health care systems and policies also make results challenging to compare between jurisdictions and health systems. Lastly, the combination of search terms was made in conjunction with three authors and a librarian; however, some relevant studies may have not been found through the keywords and MeSH terms used. However, this review has many strengths. We used a rigorous methodology, the review articles used were double screened by two authors and any conflicts were reviewed by a third author. We also feel that restricting studies to those using a validated screening and assessment tool would enable a more accurate estimation of the true effect.

## 5. Conclusions

To our knowledge, this is the first synthesis of this topic and can be used to inform future interventions and advocacy efforts for people living in long-term care. Overall, this review suggests that there is a high prevalence and risk of malnutrition in those with dementia living in long-term care facilities. Given the high prevalence, the use of validated nutrition assessment tools to assess malnutrition is strongly recommended and suggests that these tools are ideally administered by dietitians to enable rapid access to appropriate medical nutrition therapy. Future research should explore the prevalence of malnutrition in other settings including long-term care institutions in the United Kingdom, North America, and Australasia, as well as in other subgroups with dementia, such as those with dysphagia and neurological conditions.

## Figures and Tables

**Table 1:** Description of included studies (n = 24).

Author, Year Country	Study Type	Tool Used for Dementia Diagnosis	Number with Dementia	Mean Age (Years)	Gender Mix (% Female)	Mean Time Since Dementia Diagnosis (Months)	Mean Time Since Admission	Nutrition Assessment Tool/Assessor	Proportion of Well Nourished/at Risk of Malnutrition/Malnourished
Arellano et al., 2004 [11] Spain	Cross sectional	MMSE	n = 63	80.1 ± 8.1	75%	Not stated	Not stated	MNA/Not stated	Well nourished: 1.5% At risk: 36.5% Malnourished: 61.9%
Bolmsjo et al., 2015 [47] Sweden	Prospective cohort	Medical records	n = 107	Not stated	Not stated	Not stated	Not stated	MNA and MNA-SF/Nurses	Well nourished: 32.7% At risk: 45.8% Malnourished: 21.5%
Bonaccorsi et al., 2015 [30] Italy	Cross sectional	GDS and Pfeiffer test	Dementia: n = 640 Cognitive impairment: n = 1089	Not stated	Not stated	Not stated	Not stated	MUST/Nurses	At risk: Severe dementia: 50.5% Severe cognitive impairment: 47.3%
Bourdel-Marchasson et al., 2009 [32] France	Cross sectional	Dietitian confirmed as part of MNA	Severe dementia or depression: n = 868 Mild dementia: n = 24	Not stated	Not stated	Not stated	Not stated	MNA and MNA-SF/Dietitian	Malnourished Mild dementia: 35.3% Severe dementia: 60.3%
Cankurtaran et al., 2013 [31] Turkey	Cross sectional	Pre-existing	n = 420	Not stated	Not stated	Not stated	Not stated	MNA and MNA-SF/Nurses	Well nourished 29.3% At risk: 47.9% Malnourished: 22.9%
Cereda et al., 2011 [49] Italy	Prospective observational study	Medical records or having the patients interviewed and physically examined	n = 154	Not stated	Not stated	Not stated	Not stated	MNA/Dietitian	Well nourished: 5.8% At risk: 61.0% Malnourished: 33.1%
Chang et al., 2011 [10] Taiwan	Cross sectional	Medical records	n = 83	81.5 ± 7.52	59%	51.1 ± 26.9	Not stated	MNA and MNA-SF/Research assistants	Well nourished 9.6% At risk: 90.4%
Elsig et al., 2015 [34] Switzerland	Cross sectional	Various tools including MMSE, CERAD, CDR, DAD, VVPAT -WMS, the Stroop test and phonemic fluency	n = 29	82.5 ± 6.3	76%	Not stated	Not stated	MNA/Not stated	Well nourished: 6.9% At risk: 62.1% Malnourished: 31.0%
El Zoghbi et al., 2014 [35] Lebanon	Cross sectional	MMSE	n = 58	Not stated	Not stated	Not stated	Not stated	MNA/Not stated	Well nourished: 25.9% At risk: 56.9% Malnourished: 17.2%
GilGregorio et al., 2003 [44] Spain	RCT	NINCDS- ADRDA	n = 99	86.5 ± 6.1	79.8%	49.1 ± 24	20.2 ± 18.8 months	MNA/Not stated	Well nourished: 14.4% At risk: 68.1% Malnourished: 17.5%
Kamo et al., 2017 [50] Japan	Prospective observational study	Medical records	n = 56	Not stated	Not stated	Not stated	Not stated	MNA-SF/Not stated	At risk or well nourished: 24.4% Malnourished: 75.6%
Keser et al., 2016 [36] Turkey	Cross sectional	Medical records	n = 57	76.0 ± 9.84	61.4%	Not stated	2.4 ± 1.3 years	MNA/Not stated	Well nourished: 22.8% At risk: 57.9% Malnourished: 19.3%
Lauque et al., 2000 [45] France	RCT	Family/legal guardians/medical record	n = 57	Not stated	Not stated	Not stated	Not stated	MNA/Researcher	Well nourished: 15.8% At risk: 45.6% Malnourished: 38.6%
Lin et al., 2017 [48] Taiwan	Prospective cohort study	DSM and NINCDS- ADRDA	n = 70	86.1 ± 4.0	Not stated	Not stated	Not stated	MNA-SF/Not stated	Well nourished: 37.1% Malnourished or at risk: 62.9%
Maltais et al., 2018 [46] France	RCT	DSM and MMSE	n = 91	Not stated	Not stated	Not stated	Not stated	MNA/Not stated	Control—Baseline Well nourished: 4.2% At risk: 76.6% Malnutrition: 19.2% Control—6 months Well nourished: 10.6% At risk: 78.7% Malnutrition: 10.6% Exercise—Baseline Well nourished: 25% Risk: 68.2% Malnutrition: 6.8% Exercise—6 months Well nourished: 31.8% Risk: 61.4% Malnourished: 6.8%
Muurinen et al., 2015 [37] Finland	Cross sectional	Medical records	n = 2379	85	78%	Not stated	Not stated	MNA/Nurses	Well nourished 9% At risk: 63% Malnourished: 28%
Salminen et al., 2019 [38] Finland	Cross sectional	Medical records	n = 1680	Not stated	Not stated	Not stated	Not stated	MNA/Nurses	Well nourished: 15.6% Risk: 65.1% Malnutrition: 19.3%
Sliwinski et al., 2013 [39] Poland	Cross sectional	GDS	n = 62	Women: 81.5 ± 6.92 Men: 74.5 ± 7.68	60%	Not stated	Not stated	MNA/Not stated	Well nourished: 28% Risk: 65% Malnourished: 7%
Suominem et al., 2004 [40] Finland	Cross sectional	MMSE	n = 2	82	100%	Not stated	Not stated	MNA-SF and PG-SGA/Nurses	Well nourished: 0% At risk: 87% Malnourished: 13%
Suominen et al., 2007 [51] Finland	Before–After study	Medical record	n = 19	85	100%	Not stated	Not stated	MNA/Nurses	Before education Well nourished: 0% At risk: 89% Malnourished: 11% After education Well nourished: 16% At risk: 63% Malnourished: 21%
Vandewoudeet al., 2019 [41] Belgium	Cross sectional	Medical record	n = 1051	Not stated	Not stated	Not stated	Not stated	MNA-SF/Nurses, GPs and other health care providers	Well nourished 18% At risk: 62% Malnourished: 20%
Wojszel et al., 2006 [52] Poland	Pre-Post study	AMTS	n = 44	Not stated	Not stated	Not stated	Not stated	MNA/Nurses	Well nourished: 8.9% At risk: 68.9% Malnutrition 22.2%
Yap et al., 2019 [42] Malaysia	Cross sectional	Mini-cog test	n = 164	Not stated	Not stated	Not stated	Not stated	MNA/Trained healthcare personnel	Well nourished: 23.2% At risk: 58.5% Malnourished: 18.3%
Ziebolz et al., 2017 [43] Germany	Cross sectional	Medical records	n = 48	Not stated	Not stated	Not stated	Not stated	MNA/Dentist	Well nourished 0% At risk: 60% Not at risk: 40%

Legend: MMSE—Mini Mental State Exam, GDS—Global Deterioration Exam, CERAD—Consortium to Establish a Registry for Alzheimer’s Disease, CDR—Clinical Dementia Rating, DAD—Disability Assessment for Dementia, NINCDS-ADRDA—National Institute of Neurological and Communicative Disorders and Stroke (NINCDS) and the Alzheimer’s Disease and Related Disorders Association (ADRDA), DSM—Diagnostic and Statistical Manual of Mental Disorders, AMTS—abbreviated mental test score, MNA—Mini Nutritional Assessment, MNA-SF—Mini Nutritional Assessment—Short Form, MUST—Malnutrition Universal Screening Tool, P-SGA—Patient Generated—Subjective Global Assessment.

## Data Availability

The data presented in this study are available upon reasonable request from the corresponding author.

## Supplementary Materials

The following supporting information can be downloaded at: https://www.mdpi.com/article/10.3390/nu15132927/s1, Table S1: search strategy; Table S2: Quality assessment of included studies.

## References

[1] World Health Organisation Dementia: A Public Health Priority. https://apps.who.int/iris/handle/10665/75263.

[2] Alzheimers Association (2022). 2022 Alzheimer’s disease facts and figures. Alzheimer Dement..

[3] Prince M., Bryce R., Albanese E., Wimo A., Ribeiro W., Ferri C.P. (2013). The global prevalence of dementia: A systematic review and metaanalysis. Alzheimer’s Dement..

[4] Prince M., Wimo A., Guerchet M., Ali G.-C., Wu Y.-T., Prina M. World Alzheimer Report 2015. The Global Impact of Dementia. An Analysis of Prevalence, Incidence, Cost & Trends. https://www.alzint.org/u/WorldAlzheimerReport2015.pdf.

[5] Chowdhary N., Barbui C., Anstey K., Kivipelto M., Barbera M., Peters R., Zheng L., Kulmala J., Stephen R., Ferri C. (2022). Reducing the Risk of Cognitive Decline and Dementia: WHO Recommendations. Front. Neurol..

[6] Cipriani C.C.G., Lucetti C., Danti S., Nuti A. (2016). Eating Behaviors and Dietary Changes in Patients With Dementia. Am. J. Alzheimer’s Dis. Dement..

[7] Hanson L.C., Ersek M., Lin F.C., Carey T.S. (2013). Outcomes of feeding problems in advanced dementia in a nursing home population. J. Am. Geriatr. Soc..

[8] Australian Institute of Health and Welfare Dementia in Australia. https://www.aihw.gov.au/reports/dementia/dementia-in-aus/contents/aged-care-and-support-services-used-by-people-with-dementia/residential-aged-care.

[9] Soeters P., Bozzetti F., Cynober L., Forbes A., Shenkin A., Sobotka L. (2017). Defining malnutrition: A plea to rethink. Clin. Nutr..

[10] Chang C.-C., Roberts B.L. (2011). Malnutrition and feeding difficulty in Taiwanese older with dementia. J. Clin. Nurs..

[11] Arellano M., Garcia-Caselles M.P., Pi-Figueras M., Miralles R., Torres R.M., Aguilera A., Cervera A.M. (2004). Clinical impact of different scores of the Mini Nutritional Assessment (MNA) in the diagnosis of malnutrition in patients with cognitive impairment. Arch. Gerontol. Geriatr..

[12] Harris D. (2005). Malnutrition screening in the elderly population. J. R. Soc. Med..

[13] Winter J., Flanagan D., McNaughton S.A., Nowson C. (2013). Nutrition screening of older people in a community general practice, using the MNA-SF. J. Nutr. Health Aging.

[14] Keller H., Vucea V., Slaughter S.E., Jager-Wittenaar H., Lengyel C., Ottery F.D., Carrier N. (2019). Prevalence of Malnutrition or Risk in Residents in Long Term Care: Comparison of Four Tools. J. Nutr. Gerontol. Geriatr..

[15] Khater M.S., Abouelezz N.F. (2011). Nutritional status in older adults with mild cognitive impairment living in elderly homes in Cairo, Egypt. J. Nutr. Health Aging.

[16] Khater M.S., Mousa S.M. (2012). Predicting falls among Egyptian nursing home residents: A 1-year longitudinal study. J. Clin. Gerontol. Geriatr..

[17] Velázquez-Alva M.C., Irigoyen-Camacho M.E., Cabrer-Rosales M.F., Lazarevich I., Arrieta-Cruz I., Gutiérrez-Juárez R., Zepeda-Zepeda M.A. (2020). Prevalence of malnutrition and depression in older adults living in nursing homes in Mexico City. Nutrients.

[18] Liu W., Chen S., Jiang F., Zhou C., Tang S. (2020). Malnutrition and Physical Frailty among Nursing Home Residents: A Cross-Sectional Study in China. J. Nutr. Health Aging.

[19] Vellas B., Guigoz Y., Garry P.J., Nourhashemi F., Bennahum D., Lauque S., Albarede J.L. (1999). The Mini Nutritional Assessment (MNA) and its use in grading the nutritional state of elderly patients. Nutrition.

[20] Jager-Wittenaar H., Ottery F.D. (2017). Assessing nutritional status in cancer. Curr. Opin. Clin. Nutr. Metab. Care.

[21] Detsky A.S., McLaughlin J.R., Baker J.P., Johnston N., Whittaker S., Mendelson R.A., Jeejeebhoy K.N. (1987). What is subjective global assessment of nutritional status?. J. Parenter Enter. Nutr..

[22] Rubenstein L.Z., Harker J.O., Salvà A., Guigoz Y., Vellas B. (2001). Screening for undernutrition in geriatric practice: Developing the short-form mini-nutritional assessment (MNA-SF). J. Gerontol. Ser. A Biol. Sci. Med. Sci..

[23] Page M.J., McKenzie J.E., Bossuyt P.M., Boutron I., Hoffmann T.C., Mulrow C.D., Shamseer L., Tetzlaff J.M., Akl E.A., Brennan S.E. (2021). The PRISMA 2020 statement: An updated guideline for reporting systematic reviews. BMJ.

[24] (2022). Covidence Systematic Review Software, Veritas Health Innovation, Melbourne, Australia. www.covidence.org.

[25] Microsoft Corporation (2022). Microsoft Excel, Office365.

[26] Handu D.M.L., Wolfram T., Ziegler P., Acosta A., Steiber A. (2016). Academy of Nutrition and Dietetics Methodology for Conducting Systematic Reviews for the Evidence Analysis Library. J. Acad. Nutr. Diet..

[27] MedCalc Software Ltd. (2021). MedCalc® Statistical Software.

[28] Jesus P., Desport J.C., Massoulard A., Villemonteix C., Baptiste A., Gindre-Poulvelarie L., Lorgueuilleux S., Javerliat V., Fraysse J.L., Preux P.M. (2012). Nutritional assessment and follow-up of residents with and without dementia in nursing homes in the Limousin region of France: A health network initiative. J. Nutr. Health Aging.

[29] Carlsson M., Haglin L., Rosendahl E., Gustafson Y. (2013). Poor nutritional status is associated with urinary tract infection among older people living in residential care facilities. J. Nutr. Health Aging.

[30] Bonaccorsi G., Collini F., Castagnoli M., Di Bari M., Cavallini M.C., Zaffarana N., Pepe P., Mugelli A., Lucenteforte E., Vannacci A. (2015). A cross-sectional survey to investigate the quality of care in Tuscan (Italy) nursing homes: The structural, process and outcome indicators of nutritional care. BMC Health Serv. Res..

[31] Cankurtaran M., Saka B., Sahin S., Varli M., Doventas A., Yavuz B.B., Halil M., Curgunlu A., Ulger Z., Tekin N. (2013). Turkish nursing homes and care homes nutritional status assessment project (THN-malnutrition). Eur. Geriatr. Med..

[32] Bourdel-Marchasson I., Rolland C., Jutand M.-A., Egea C., Baratchart B., Barberger-Gateau P. (2009). Undernutrition in geriatric institutions in South-West France: Policies and risk factors. Nutrition.

[33] Boström A.-M., Van Soest D., Kolewaski B., Milke D.L., Estabrooks C.A. (2011). Nutrition Status Among Residents Living in a Veterans’ Long-Term Care Facility in Western Canada: A Pilot Study. J. Am. Med. Dir. Assoc..

[34] Elsig F., Schimmel M., Duvernay E., Giannelli S.V., Graf C.E., Carlier S., Herrmann F.R., Michel J.P., Gold G., Zekry D. (2015). Tooth loss, chewing efficiency and cognitive impairment in geriatric patients. Gerodontology.

[35] El Zoghbi M., Boulos C., Awada S., Rachidi S., Al-Hajje A., Bawab W., Saleh N., Salameh P. (2014). Prevalence of malnutrition and its correlates in older adults living in long stay institutions situated in Beirut, Lebanon. J. Res. Health Sci..

[36] Keser A., Yildirim F. (2016). Evaluation of the relationship between nutritional status and quality of life among nursing home residents with alzheimer’s disease. Improv. Qual. Life Dement. Patients Prog. Detect. Treat. Care.

[37] Muurinen S., Savikko N., Soini H., Suominen M., Pitkälä K. (2015). Nutrition and psychological well-being among long-term care residents with dementia. J. Nutr. Health Aging.

[38] Salminen K.S., Suominen M.H., Soini H., Kautiainen H., Savikko N., Saarela R.K.T., Muurinen S., Pitkala K.H. (2019). Associations Between Nutritional Status and Health-Related Quality of Life Among Long-Term Care Residents in Helsinki. J. Nutr. Health Aging.

[39] Sliwinski Z., Matlok M., Starczynska M., Makara-Studzinska M. (2013). Mental and physical performance of dementia patients in long-term residential care. Med. Stud. Stud. Med..

[40] Suominem M., Laine T., Routasalo P., Pitkala K.H., Rasanen L. (2004). Nutrient content of served food, nutrient intake and nutritional status of residents with dementia in a finnish nursing home. J. Nutr. Health Aging.

[41] Vandewoude M.F.J., van Wijngaarden J.P., De Maesschalck L., Luiking Y.C., Van Gossum A. (2019). The prevalence and health burden of malnutrition in Belgian older people in the community or residing in nursing homes: Results of the NutriAction II study. Aging Clin. Exp. Res..

[42] Yap S.F., Boo N.Y., Shenoy P.D., Liew S.F., Woo L.F., Choo P.Y., Leong P.P., Hatta N.M. (2019). Nutritional status of elderly residents of long-term care homes in Klang Valley, Malaysia: A cross-sectional study. Asian J. Gerontol. Geriatr..

[43] Ziebolz D., Werner C., Schmalz G., Nitschke I., Haak R., Mausberg R.F., Chenot J.-F. (2017). Oral Health and nutritional status in nursing home residents-results of an explorative cross-sectional pilot study. BMC Geriatr..

[44] Gil Gregorio P., Ramirez Diaz S.P., Ribera Casado J.M., Tobaruela J.L., Neira R., Medina J., González P., Navarro C., Robledillo R., Moreno J. (2003). Dementia and nutrition. Intervention study in institutionalized patients with Alzheimer Disease. J. Nutr. Health Aging.

[45] Lauque S., Arnaud-Battandier F., Mansourian R., Guigoz Y., Paintin M., Nourhashemi F., Vellas B. (2000). Protein-energy oral supplementation in malnourished nursing-home residents. A controlled trial. Age Ageing.

[46] Maltais M., Rolland Y., Hay P.E., Armaingaud D., Cestac P., Rouch L., Barreto P.D. (2018). The Effect of Exercise and Social Activity Interventions on Nutritional Status in Older Adults with Dementia Living in Nursing Homes: A Randomised Controlled Trial. J. Nutr. Health Aging.

[47] Bolmsjo B.B., Jakobsson U., Molstad S., Ostgren C.J., Midlov P. (2015). The nutritional situation in Swedish nursing homes—A longitudinal study. Arch. Gerontol. Geriatr..

[48] Lin C.-S., Lin S.-Y., Chou M.-Y., Chen L.-Y., Wang K.-Y., Chen L.-K., Lin Y.-T., Loh C.-H. (2017). Hospitalization and associated factors in people with Alzheimer’s disease residing in a long-term care facility in southern Taiwan. Geriatr. Gerontol. Int..

[49] Cereda E., Pedrolli C., Zagami A., Vanotti A., Piffer S., Opizzi A., Rondanelli M., Caccialanza R. (2011). Nutritional screening and mortality in newly institutionalised elderly: A comparison between the Geriatric Nutritional Risk Index and the Mini Nutritional Assessment. Clin. Nutr..

[50] Kamo T., Takayama K., Ishii H., Suzuki K., Eguchi K., Nishida Y. (2017). Coexisting severe frailty and malnutrition predict mortality among the oldest old in nursing homes: A 1-year prospective study. Arch. Gerontol. Geriatr..

[51] Suominen M.H., Kivisto S.M., Pitkala K.H. (2007). The effects of nutrition education on professionals’ practice and on the nutrition of aged residents in dementia wards. Eur. J. Clin. Nutr..

[52] Wojszel Z.B. (2006). Determinants of nutritional status of older people in long-term care settings on the example of the nursing home in Białystok. Adv. Med. Sci..

[53] MapChart.com World Map—Simple: Create a Custom Map. MapChart. https://www.mapchart.net/world.html.

[54] Kaiser M.J., Bauer J.M., Ramsch C., Uter W., Guigoz Y., Cederholm T., Thomas D.R., Anthony P., Charlton K.E., Maggio M. (2009). Validation of the Mini Nutritional Assessment short-form (MNA-SF): A practical tool for identification of nutritional status. J. Nutr. Health Aging.

[55] Stratton R.J., Hackston A., Longmore D., Dixon R., Price S., Stroud M., King C., Elia M. (2004). Malnutrition in hospital outpatients and inpatients: Prevalence, concurrent validity and ease of use of the ‘malnutrition universal screening tool’ (‘MUST’) for adults. Br. J. Nutr..

[56] Plassman B.L., Langa K.M., McCammon R.J., Fisher G.G., Potter G.G., Burke J.R., Steffens D.C., Foster N.L., Giordani B., Unverzagt F.W. (2011). Incidence of dementia and cognitive impairment, not dementia in the United States. Ann. Neurol..

[57] Prince M., Knapp M., Guerchet M., McCrone P., Prina M., Comas Herrera A., Wittenberg A., Adelaja R., Hu B., King B. (2014). Dementia UK Update.

[58] Robb L., Walsh C.M., Nel M., Nel A., Odendaal H., van Aardt R. (2017). Malnutrition in the elderly residing in long-term care facilities: A cross sectional survey using the Mini Nutritional Assessment (MNA®) screening tool. S. Afr. J. Clin. Nutr..

[59] Yalcin A., Silay K. (2017). Sarcopenia and health-related quality of life in turkish nursing home residents: A cross-sectional study. Asian J. Gerontol. Geriatr..

[60] Eisenmann Y., Golla H., Schmidt H., Voltz R., Perrar K.M. (2020). Palliative Care in Advanced Dementia. Front. Psychiatry.

[61] Cereda E., Pedrolli C., Klersy C., Bonardi C., Quarleri L., Cappello S., Turri A., Rondanelli M., Caccialanza R. (2016). Nutritional status in older persons according to healthcare setting: A systematic review and meta-analysis of prevalence data using MNA (R). Clin. Nutr..

[62] Donini L.M., Neri B., De Chiara S., Poggiogalle E., Muscaritoli M. (2013). Nutritional Care in a Nursing Home in Italy. PLoS ONE.

[63] Kabir Z.N., Ferdous T., Cederholm T., Khanam M.A., Streatfied K., Wahlin A. (2006). Mini Nutritional Assessment of rural elderly people in Bangladesh: The impact of demographic, socio-economic and health factors. Public Health Nutr..

[64] Crichton M., Craven D., Mackay H., Marx W., De Van Der Schueren M., Marshall S. (2018). A systematic review, meta-analysis and meta-regression of the prevalence of protein-energy malnutrition: Associations with geographical region and sex. Age Ageing.

[65] Tom S.E., Hubbard R.A., Crane P.K., Haneuse S.J., Bowen J., McCormick W.C., McCurry S., Larson E.B. (2015). Characterization of dementia and Alzheimer’s disease in an older population: Updated incidence and life expectancy with and without dementia. Am. J. Public Health.

[66] Australian Institute of Health and Welfare People Using Aged Care. https://www.gen-agedcaredata.gov.au/Topics/People-using-aged-care.

[67] Doorduijn A.S., de van der Schueren M.A.E., van de Rest O., de Leeuw F.A., Hendriksen H.M.A., Teunissen C.E., Scheltens P., van der Flier W.M., Visser M. (2020). Energy intake and expenditure in patients with Alzheimer’s disease and mild cognitive impairment: The NUDAD project. Alzheimers Res..

[68] Spencer J.C., Damanik R., Ho M.-H., Montayre J., Traynor V., Chang C.-C., Chang H.-C. (2021). Review of Food Intake Difficulty Assessment Tools for People with Dementia. West. J. Nurs. Res..

[69] Chang C.-C., Lin Y.-F., Chiu C.-H., Liao Y.-M., Ho M.-H., Lin Y.-K., Chou K.-R., Liu M.F. (2017). Prevalence and factors associated with food intake difficulties among residents with dementia. PLoS ONE.

[70] Namasivayam A.M., Steele C.M. (2015). Malnutrition and Dysphagia in Long-Term Care: A Systematic Review. J. Nutr. Gerontol. Geriatr..

[71] KS S., MH S., H K., HM R., KH P. (2019). Energy Intake and Severity of Dementia Are Both Associated with Health-Related Quality of Life among Older Long-Term Care Residents. Nutrients.

[72] Kondrup J., Allison S.P., Elia M., Vellas B., Plauth M. (2003). ESPEN Guidelines for Nutrition Screening 2002. Clin. Nutr..

[73] Kellett J., Kyle G., Itsiopoulos C., Naunton M. (2016). Nutrition screening practices amongst australian Residential Aged Care Facilities. J. Nutr. Health Aging.

[74] Baek M.-H., Heo Y.-R. (2015). Evaluation of the efficacy of nutritional screening tools to predict malnutrition in the elderly at a geriatric care hospital. Nutr. Res. Pract..

[75] Sánchez-Rodríguez D., Annweiler C., Ronquillo-Moreno N., Tortosa-Rodríguez A., Guillén-Solà A., Vázquez-Ibar O., Escalada F., Muniesa J.M., Marco E. (2018). Clinical application of the basic definition of malnutrition proposed by the European Society for Clinical Nutrition and Metabolism (ESPEN): Comparison with classical tools in geriatric care. Arch. Gerontol. Geriatr..

[76] Diekmann R., Winning K., Uter W., Kaiser M., Sieber C., Volkert D., Bauer J. (2013). Screening for malnutrition among nursing home residents—A comparative analysis of the Mini Nutritional Assessment, the Nutritional Risk Screening, and the Malnutrition Universal Screening Tool. J. Nutr. Health Aging.

[77] Gorji H.A., Alikhani M., Mohseni M., Moradi-Joo M., Ziaiifar H., Moosavi A. (2017). The prevalence of malnutrition in iranian elderly: A review article. Iran. J. Public Health.

[78] Kokura Y., Momosaki R. (2022). Prevalence of Malnutrition Assessed by the GLIM Criteria and Association with Activities of Daily Living in Older Residents in an Integrated Facility for Medical and Long-Term Care. Nutrients.

[79] Bajeux E., Corvol A., Somme D. (2021). Integrated Care for Older People in France in 2020: Findings, Challenges, and Prospects. Int. J. Integr. Care.

[80] Grosshauser F.J., Kiesswetter E., Torbahn G., Sieber C.C., Volkert D. (2021). Reasons for and against Nutritional Interventions. An Exploration in the Nursing Home Setting. Geriatrics.

[81] Suominen M.H., Sandelin E., Soini H., Pitkala K.H. (2009). How well do nurses recognize malnutrition in elderly patients?. Eur. J. Clin. Nutr..

[82] Beattie E., O’Reilly M., Strange E., Franklin S., Isenring E. (2014). How much do residential aged care staff members know about the nutritional needs of residents?. Int. J. Older People Nurs..

