# Supplementary material for: Prevalence of Malnutrition in People with Dementia in Long-Term Care: A Systematic Review and Meta-Analysis
Source: Nutrients. 2023 Jun 28;15(13):2927. doi: 10.3390/nu15132927 (PMC10343750; doi:10.3390/nu15132927)
Supplement: Supplementary file 1 [file nutrients-15-02927-s001.zip › nutrients-2463496-supplementary.pdf]

Supplementary Table S1. Search strategy

**Scopus**<br>TITLE-ABS-KEY(Malnutrition OR malnourished OR undernutrition OR undernourished OR "protein-energy malnutrition" OR sarcopenia OR "risk of malnutrition" OR "weight loss" OR "prevalence of malnutrition" OR "nutritional status") AND TITLE-ABS-KEY("subjective global assessment" OR SGA OR "patient generated subjective global assessment" OR "PG-SGA" OR "mini nutritional assessment" OR MNA OR "mini nutritional assessment short form" OR MNA-SF OR "nutrition assessment" OR "geriatric assessment" OR "malnutrition screening") AND TITLE-ABS-KEY("residential aged care" OR "aged care" OR "long term care" OR "nursing home" OR "home for the ag*" OR "housing for the elderly" OR "skilled nursing facility" OR "assisted living" OR "residential care" OR "geriatric institution*")<br>*Results*: 1,142
**Web of Science – Core Collection**<br><br>((TS=(Malnutrition OR malnourished OR undernutrition OR undernourished OR "protein-energy malnutrition" OR sarcopenia OR "risk of malnutrition" OR "weight loss" OR "prevalence of malnutrition" OR "nutritional status")) AND TS=("subjective global assessment" OR SGA OR "patient generated subjective global assessment" OR "PG-SGA" OR "mini nutritional assessment" OR MNA OR "mini nutritional assessment short form" OR MNA-SF OR "nutrition assessment" OR "geriatric assessment" OR "malnutrition screening")) AND TS=("residential aged care" OR "aged care" OR "long term care" OR "nursing home" OR "home for the ag*" OR "housing for the elderly" OR "skilled nursing facility" OR "assisted living" OR "residential care" OR "geriatric institution*")<br>*Results*: 501
**CINAHL Plus with full text**<br><br>S1<br>MH (Malnutrition OR malnourished OR undernutrition OR undernourished OR "protein-energy malnutrition" OR sarcopenia OR "risk of malnutrition" OR "weight loss" OR "prevalence of malnutrition" OR "nutritional status") OR TI (Malnutrition OR malnourished OR undernutrition OR undernourished OR "protein-energy malnutrition" OR sarcopenia OR "risk of malnutrition" OR "weight loss" OR "prevalence of malnutrition" OR "nutritional status") OR AB (Malnutrition OR malnourished OR undernutrition OR undernourished OR "protein-energy malnutrition" OR sarcopenia OR "risk of malnutrition" OR "weight loss" OR "prevalence of malnutrition" OR "nutritional status")<br><br>S2<br>MH ("subjective global assessment" OR SGA OR "patient generated subjective global assessment" OR "PG-SGA" OR "mini nutritional assessment" OR MNA OR "mini nutritional assessment short form" OR MNA-SF OR "nutrition assessment" OR "geriatric assessment" OR "malnutrition screening") OR TI ("subjective global assessment" OR SGA OR "patient generated subjective global assessment" OR "PG-SGA" OR "mini nutritional assessment" OR MNA OR "mini nutritional assessment short form" OR MNA-SF OR "nutrition assessment" OR "geriatric assessment" OR "malnutrition screening") OR AB ("subjective global assessment" OR SGA OR "patient generated subjective global assessment" OR "PG-SGA" OR "mini nutritional assessment" OR MNA OR "mini nutritional assessment short form" OR MNA-SF OR "nutrition assessment" OR "geriatric assessment" OR "malnutrition screening")<br><br>S3<br>MH ("residential aged care" OR "aged care" OR "long term care" OR "nursing home" OR "home for the ag*" OR "housing for the elderly" OR "skilled nursing facility" OR "assisted living" OR "residential care" OR "geriatric institution*") OR TI ("residential aged care" OR "aged care" OR "long term care" OR "nursing home" OR "home for the ag*" OR "housing for the elderly" OR "skilled nursing facility" OR "assisted living" OR "residential care" OR "geriatric institution*") OR AB ("residential aged care" OR "aged care" OR "long term care" OR "nursing home" OR "home for the ag*" OR "housing for the elderly" OR "skilled nursing facility" OR "assisted living" OR "residential care" OR "geriatric institution*")<br><br>Together – search with AND:<br>S1 AND S2 AND S3

Results: 320
**MEDLINE**<br><br>S1<br>MH (Malnutrition OR malnourished OR undernutrition OR undernourished OR “protein-energy malnutrition” OR sarcopenia OR “risk of malnutrition” OR “weight loss” OR “prevalence of malnutrition” OR “nutritional status”) OR AB (Malnutrition OR malnourished OR undernutrition OR undernourished OR “protein-energy malnutrition” OR sarcopenia OR “risk of malnutrition” OR “weight loss” OR “prevalence of malnutrition” OR “nutritional status”) OR TI (Malnutrition OR malnourished OR undernutrition OR undernourished OR “protein-energy malnutrition” OR sarcopenia OR “risk of malnutrition” OR “weight loss” OR “prevalence of malnutrition” OR “nutritional status”)<br><br>S2<br>MH (“subjective global assessment” OR SGA OR “patient generated subjective global assessment” OR “PG-SGA” OR “mini nutritional assessment” OR MNA OR “mini nutritional assessment short form” OR MNA-SF OR “nutrition assessment” OR “geriatric assessment” OR “malnutrition screening”) OR AB (“subjective global assessment” OR SGA OR “patient generated subjective global assessment” OR “PG-SGA” OR “mini nutritional assessment” OR MNA OR “mini nutritional assessment short form” OR MNA-SF OR “nutrition assessment” OR “geriatric assessment” OR “malnutrition screening”) OR TI (“subjective global assessment” OR SGA OR “patient generated subjective global assessment” OR “PG-SGA” OR “mini nutritional assessment” OR MNA OR “mini nutritional assessment short form” OR MNA-SF OR “nutrition assessment” OR “geriatric assessment” OR “malnutrition screening”)<br><br>S3<br>MH (“residential aged care” OR “aged care” OR “long term care” OR “nursing home” OR “home for the ag*” OR “housing for the elderly” OR “skilled nursing facility” OR “assisted living” OR “residential care” OR “geriatric institution*”) OR AB (“residential aged care” OR “aged care” OR “long term care” OR “nursing home” OR “home for the ag*” OR “housing for the elderly” OR “skilled nursing facility” OR “assisted living” OR “residential care” OR “geriatric institution*”) OR TI (“residential aged care” OR “aged care” OR “long term care” OR “nursing home” OR “home for the ag*” OR “housing for the elderly” OR “skilled nursing facility” OR “assisted living” OR “residential care” OR “geriatric institution*”)<br><br>S1 AND S2 AND S3<br>Results: 618

Supplementary Table S2: Quality assessment of included studies [26]

Author, Year	Class	Quality Rating (*)	Q1	Q2	Q3	Q4	Q5	Q6	Q7	Q8	Q9	Q10
Arellano 2004	D	Neutral	Y	U/C	N/A	N	N/A	N	Y	N	N	U/C
Bolmsjo 2015	B	Positive	Y	Y	Y	Y	N/A	Y	Y	Y	Y	Y
Bonaccorsi 2015	D	Positive	Y	Y	Y	N/A	N/A	Y	Y	Y	Y	Y
Bourdel-Marchasson 2009	D	Neutral	Y	U/C	Y	N/A	N/A	U/C	Y	Y	Y	U/C
Cankurtaran 2013	D	Positive	Y	Y	Y	N/A	N/A	Y	Y	Y	Y	Y
Cereda 2011	B	Positive	Y	Y	Y	Y	N/A	Y	Y	Y	Y	Y
Chang 2011	D	Positive	Y	Y	Y	N/A	N/A	Y	Y	Y	Y	Y
Elsig 2015	D	Positive	Y	Y	Y	N/A	N/A	Y	Y	Y	Y	Y
Elzoghbi 2014	D	Positive	Y	Y	Y	Y	N/A	Y	Y	Y	Y	Y
GilGregorio 2003	A	Neutral	Y	Y	U/C	N	N/A	N	Y	Y	N	U/C
Kamo 2017	B	Positive	Y	Y	U/C	Y	N/A	No	Y	Y	Y	U/C
Keser 2016	D	Positive	Y	Y	Y	N/A	N/A	Y	Y	Y	Y	U/C
Lauque 2000	A	Positive	Y	Y	Y	Y	N	Y	Y	Y	N	U/C
Lin 2017	B	Neutral	Y	U/C	N/A	N	N/A	N	Y	Y	U/C	U/C
Maltais 2018	A	Positive	Y	Y	Y	Y	Y	Y	Y	Y	Y	U/C
Muurinen 2015	D	Positive	Y	Y	Y	N/A	N/A	N/A	Y	Y	Y	Y
Salminen 2019	D	Positive	Y	Y	N/A	U/C	N/A	N/A	Y	Y	Y	Y
Sliwinski 2013	D	Neutral	Y	U/C	N/A	N/A	N/A	N/A	Y	Y	Y	Y
Suominem 2004	D	Positive	Y	Y	N/A	N/A	N/A	N/A	Y	Y	Y	Y
Suominen 2007	D	Neutral	Y	U/C	N/A	U/C	U/C	N	U/C	Y	Y	Y
Vandewoude 2019	D	Positive	Y	Y	N/A	U/C	U/C	N/A	Y	Y	Y	Y
Wojszel 2006	D	Positive	Y	U/C	N/A	Y	N	N/A	Y	Y	Y	Y
Yap 2019	D	Positive	Y	Y	N/A	Y	U/C	N/A	Y	Y	Y	Y
Ziebolz 2017	D	Positive	Y	Y	N/A	Y	Y	N/A	Y	Y	Y	Y

Legend: Y – Yes, N – No, N/A – Not applicable, U/C – Unclear, * – Indicates hierarchy and class of studies Q1: Was the research question clearly stated?; Q2: Was the selection of study subjects/patients free from bias?; Q3; Were study groups comparable?; Was method of handling withdrawals described?; Q5: Was blinding used to prevent introduction of bias?; Q6: Were intervention/therapeutic regimes/exposure factor or procedure and any comparison(s) described in detail? Were intervening factors described?; Q7: Were outcomes clearly defined and the measurements valid and reliable?; Q8: Was the statistical analysis appropriate for the study design and type of outcome indicators?; Q9: Are conclusions supported by results with biases and limitations taken into consideration?; Q10: Is bias due to study's funding or sponsorship unlikely?
